# Risk-Adjusted Inpatient Falls as Indicators of Health System Performance During the COVID-19 Pandemic

**DOI:** 10.3390/healthcare14030358

**Published:** 2026-01-30

**Authors:** Masae Satoh, Toko Nakahori, Tomoko Shimada

**Affiliations:** 1 Department of Nursing, Graduate School of Medicine, Yokohama City University, 3-9 Fukuura, Kanazawa-ku, Yokohama 236-0004, Japan; t226612b@yokohama-cu.ac.jp; 2 Nursing Department, Yokohama City University Hospital, Yokohama City University, 3-9 Fukuura, Kanazawa-ku, Yokohama 236-0004, Japan; s_tomoko@yokohama-cu.ac.jp

**Keywords:** patient safety, inpatient falls, risk adjustment, benchmarking, health system performance, acute care, nursing, seasonality, system stress

## Abstract

**Highlights:**

**What are the main findings?**

- Risk-adjusted inpatient fall indicators changed systematically during periods of monthly and large-scale system stress.
- Risk-adjusted safety performance improved over time despite increasing patient risk.

**What are the implications of the main findings?**

- Risk-adjusted fall monitoring supports the evaluation of hospital safety performance during seasonal changes and system-wide disruptions.
- Benchmarking fall outcomes provides a practical approach for safety surveillance in acute care settings.

**Abstract:**

**Background/Objectives:** Inpatient falls are widely used patient safety indicators, yet their behavior under periods of large-scale health system stress remains insufficiently understood. This study aimed to evaluate whether risk-adjusted inpatient fall indicators can capture changes in hospital safety performance during such periods, using a prolonged system disruption as an empirical context. The study period was a priori divided into three phases (pre-pandemic, initial pandemic, and later pandemic) according to changes in COVID-19 admission burden and system stress intensity. **Methods:** We conducted a retrospective observational time-series analysis using daily inpatient fall events and census data from a Japanese acute care hospital between December 2018 and March 2023 (50,140 inpatients; 962 falls). Expected fall rates were estimated using a validated pre-disruption prediction model, and observed/expected (O/E) ratios were calculated to assess risk-adjusted safety performance. Ordinary least squares regression models adjusted for calendar month and seasonal Fourier terms were used to examine temporal associations between fall outcomes and indicators of hospital-level system burden. **Results:** Both observed and expected fall rates increased during the study period, whereas O/E ratios declined only in the later phase, indicating improvement in risk-adjusted safety performance despite rising intrinsic patient risk. Seasonal patterns in fall outcomes were disrupted during the early phase of system stress but re-emerged over time. Associations between system burden indicators and fall outcomes were most pronounced in the early phase and attenuated in later phases. **Conclusions:** Risk-adjusted monitoring of inpatient falls provides insight into dynamic changes in hospital safety performance during periods of large-scale system stress and subsequent adaptation. This indicator can also be interpreted as a benchmarking scale for future month-to-month and seasonal safety surveillance beyond crisis contexts.

## 1. Introduction

Inpatient falls are among the most common adverse events in acute care hospitals and are associated with fractures, loss of independence in activities of daily living, and prolonged hospitalization [1,2]. Because fall events are closely linked to nursing surveillance, care processes, and ward-level organization, inpatient fall rates are widely used as patient safety indicators in hospital quality monitoring [3,4].

Extensive research has focused on identifying patient-level risk factors for inpatient falls and developing predictive models to support targeted prevention strategies [5,6,7]. While these efforts have advanced individual risk stratification, they provide limited insight into how fall outcomes function as indicators of hospital-level safety performance over time.

Periods of large-scale system stress—such as the COVID-19 pandemic—can substantially alter staffing patterns, workflows, and care priorities in acute care settings [8,9,10]. During the COVID-19 pandemic, hospitals experienced marked fluctuations in patient volume, infection control demands, and staffing availability, all of which may have affected fall prevention practices. Although several studies have reported changes in fall incidence during the pandemic, most have relied on crude fall rates without accounting for underlying patient risk [11,12].

Risk-adjusted monitoring using observed-to-expected (O/E) ratios has been proposed as a more precise indicator of care quality and safety performance [13,14]. However, to date, no temporal analyses have examined the association between O/E ratios for inpatient falls and hospital-level COVID-19 burden. Moreover, since system-level exhaustion and operational disruptions may not manifest immediately, the potential time-lagged relationships between changes in COVID-19 admissions and subsequent variations in fall outcomes remain largely unexplored.

In Japan, patients with COVID-19 were first admitted to acute care hospitals in February 2020, followed by repeated declarations of a national state of emergency through September 2021, a period characterized by substantial strain on hospital systems and sustained changes in clinical operations [15,16]. After this period, social and healthcare practices gradually returned to pre-pandemic conditions.

Therefore, this study aimed to describe temporal changes in observed and expected fall rates—and in observed-to-expected (O/E) ratios—in general acute care wards during the COVID-19 pandemic and to assess their associations with new COVID-19 admissions using lag analysis. Expected fall rates were estimated using a pre-pandemic fall prediction model previously developed and validated in an acute care setting [17], allowing us to evaluate how hospital safety performance deviated from pre-pandemic standards.

## 2. Materials and Methods

### 2.1. Aim

This study aimed to evaluate longitudinal changes in inpatient fall outcomes using a risk-standardized benchmarking approach under COVID-19 burden. Specifically, the objectives were to (1) characterize temporal patterns in observed fall rates, expected fall rates, and O/E ratios across distinct phases of system disruption and (2) explore temporal associations between indicators of system burden and fall outcomes.

### 2.2. Design

This study employed a retrospective, observational time-series design, incorporating risk-standardized benchmarking, to evaluate inpatient safety performance under system-level stress. This study was reported in accordance with the Strengthening the Reporting of Observational Studies in Epidemiology (STROBE) guidelines.

### 2.3. Setting and Data

**Figure 1:**
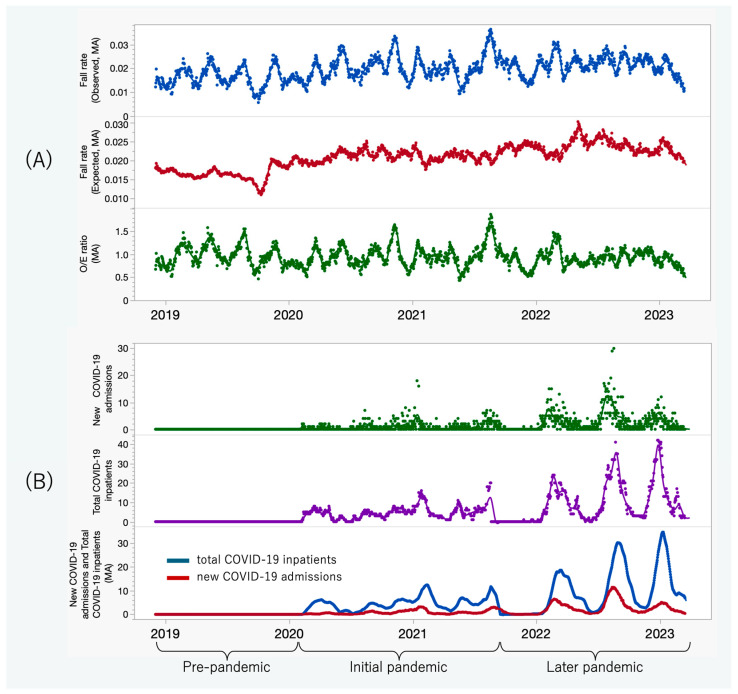
Temporal trends in fall-related outcomes and COVID-19 burden during the study period. (**A**) The 28-day moving averages of observed fall rates, expected fall rates, and observed-to-expected ratios. (**B**) Daily counts of new COVID-19 admissions and total COVID-19 inpatients, with respective 28-day moving averages. Time periods: pre-pandemic (1 December 2018–8 February 2020); initial pandemic (9 February 2020–30 September 2021); later pandemic (1 October 2021–31 March 2023).

This study was conducted at a large acute care hospital in Japan using daily inpatient fall events and census data collected between 1 December 2018 and 31 March 2023. A 26-item fall risk assessment score routinely administered upon admission was used for baseline risk evaluation [17]. Fall events were obtained from the hospital’s incident reporting system, and inpatient census data were extracted from administrative records. For analytical purposes, the study period was divided into three phases: a pre-pandemic period, an initial pandemic period during which national states of emergency were declared, and a later pandemic period without active emergency declarations. All analyses were conducted across these three periods, as illustrated in Figure 1.

Patients aged 16 years or older who were admitted to general wards and discharged during the study period were included. Patients admitted to intensive care, psychiatric, pediatric, or designated infectious disease units were excluded. In total, 50,140 inpatients and 962 fall events met the inclusion criteria. To characterize system-level burden, daily counts of new COVID-19 admissions and the total number of hospitalized patients with COVID-19 were recorded as indicators of acute and sustained operational pressure, respectively.

### 2.4. Measures

The primary outcome was the observed fall rate, summarized using 28-day moving averages to reduce short-term variability. This window length was selected to provide a stable representation of temporal trends while preserving the interpretability of longitudinal changes.

Expected fall rates were estimated using a validated pre-pandemic logistic regression model [17]. The fixed six-factor model comprised the following binary predictors: age ≥65 years, impaired extremities, muscle weakness, requirement for mobility assistance, unstable gait, and psychotropic medication use. The model generated individual fall probabilities based on regression coefficients rather than a point-based scoring system.

The regression equation was specified as

Logit(*p*) = −3.048 + 0.339 (Age ≥ 65) + 0.340 (Impaired extremities) + 0.492 (Muscle weakness) + 0.418 (Mobility assistance) + 0.466 (Unstable gait) + 0.342 (Psychotropic medication use),

where *p* denotes the probability of an inpatient fall, and *p* = 1/[1 + exp(–Logit(*p*))].

In the original validation cohort, the model demonstrated acceptable discriminative performance, with an area under the receiver operating characteristic curve of approximately 0.78. Model coefficients were not recalibrated during the pandemic period in order to preserve a fixed pre-disruption risk standard.

The O/E ratio, calculated as the observed fall rate divided by the expected fall rate, served as the primary risk-standardized indicator of safety performance, allowing deviations from unity to reflect changes in system-level safety performance rather than shifts in individual risk composition.

System burden was represented using two indicators: (1) the daily number of new COVID-19 admissions, reflecting acute system pressure, and (2) the total number of hospitalized patients with COVID-19, representing sustained operational burden. These indicators were used to contextualize safety performance under fluctuating care demands rather than as causal determinants of fall events.

### 2.5. Statistical Analysis

Ordinary least squares (OLS) time-series regression models were fitted separately for each phase of system disruption to examine associations between system burden indicators and fall outcomes. This phase-stratified approach enabled a direct comparison of association patterns under differing levels of system strain. Calendar-month fixed effects and Fourier seasonal terms were included to account for policy-related and cyclic variation.

OLS regression was selected to provide a transparent and interpretable framework for estimating average temporal associations, consistent with the study’s descriptive and exploratory objectives rather than predictive forecasting. To mitigate short-term autocorrelation, fall-related outcomes and system burden indicators were smoothed using 28-day moving averages, and residual diagnostics were inspected to assess serial dependence.

Additional exploratory analyses using lagged indicators ranging from 0 to 28 days were conducted to support the interpretation of temporal patterns in fall outcomes. These lag analyses were theoretically motivated by the assumption that system strain may influence safety outcomes with a delayed effect due to cumulative disruptions in staffing and care processes. These analyses were not intended for causal inference.

Differences in regression coefficients across phases were interpreted as indicative of changes in the strength of association under varying system conditions rather than as formal interaction effects. All statistical analyses were performed using JMP Student Edition version 18.2.0 (SAS Institute, Cary, NC, USA). A two-sided *p*-value of <0.05 was considered statistically significant.

### 2.6. Ethical Considerations

This study used routinely collected, de-identified data and was conducted in accordance with institutional and national ethical standards for research. This study was approved by the Ethics Committee of Epidemiological Research of Yokohama City University (approval number: F230800007; approval date: 15 September 2023). Study details were publicly disclosed on the hospital website to allow eligible individuals to opt out. All data were anonymized prior to analysis and securely stored on password-protected servers with restricted access.

## 3. Results

Overall, inpatient fall outcomes exhibited distinct temporal patterns across pandemic phases. While observed and expected fall rates increased over time, risk-adjusted O/E ratios showed phase-specific shifts, and associations with COVID-19 burden varied between the initial and later pandemic periods. These patterns are described below, beginning with descriptive trends followed by phase-specific regression results.

### 3.1. Temporal Patterns in Fall Indicators Across Pandemic Phases

Across the study period, observed fall rates showed greater short-term variability than expected fall rates, while O/E ratios remained centered around unity with phase-specific shifts.

Figure 1A depicts the 28-day moving averages of observed fall rates, expected fall rates, and O/E ratios. Observed fall rates exhibited pronounced short-term variability, particularly during the initial pandemic phase, whereas fluctuations stabilized in the later phase. Expected fall rates increased gradually and consistently throughout the study period. As expected from their construction, O/E ratios closely followed observed fall dynamics, although with a lower magnitude than raw incidence.

Table 1 presents least-squares means (±SEM) based on 28-day averaged data, adjusted for calendar month and pandemic phase. The mean observed fall rate increased from 1.63% in the pre-pandemic phase to 2.05% in the initial phase and 2.12% in the later phase. Expected fall rates demonstrated a similar upward trend (1.68%, 2.12%, and 2.36%, respectively). The O/E ratio remained near unity during the pre-pandemic (0.98) and initial (0.97) phases before declining to 0.90 in the later phase, indicating improved risk-adjusted safety performance despite rising intrinsic patient risk.

### 3.2. System Pressure Dynamics: COVID-19 Admission Patterns

COVID-19 system burden exhibited distinct short-term and cumulative dynamics over time.

Figure 1B depicts daily new COVID-19 admissions alongside the total number of hospitalized patients with COVID-19. Peaks in total inpatient census consistently followed surges in new admissions, reflecting the accumulation of system pressure over subsequent days rather than an immediate surge. This temporal pattern provides important context for interpreting subsequent safety outcomes.

### 3.3. Phase-Specific Regression Results: Seasonal and COVID-19-Related Effects

Associations between fall indicators and COVID-19 burden differed by pandemic phase and outcome type.

**Figure 2:**
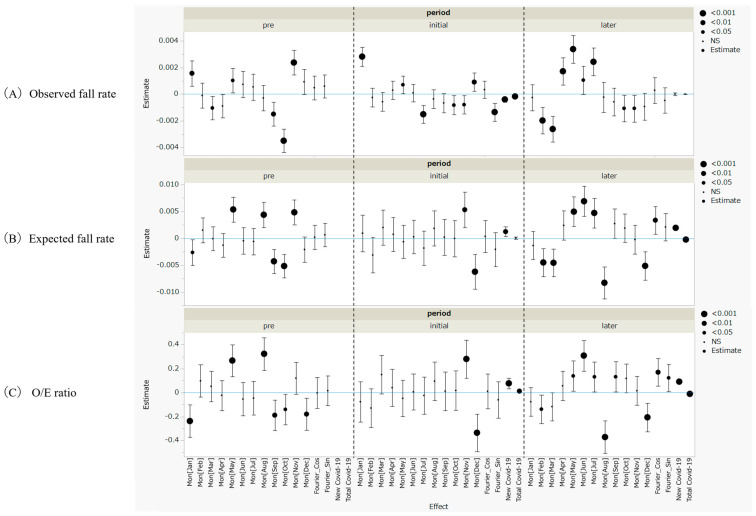
Monthly and COVID-19-related effects on fall rates and O/E ratios across pandemic phases. Estimates and 95% confidence intervals were sourced from ordinary least squares regression models, including month effects, Fourier terms, and COVID-19 related-variables. Significance levels are indicated by symbol size. New COVID-19 and Total COVID-19 represent new COVID-19 admissions and total COVID-19 inpatients, respectively.

Figure 2 summarizes the ordinary least squares (OLS) regression estimates; full coefficient estimates are provided in Supplementary Table S1. Table 2 presents regression coefficients for COVID-19-related variables.

#### 3.3.1. Observed Fall Rate

Seasonal oscillations were pronounced during the pre-pandemic phase, as reflected by significant monthly peaks and troughs (Figure 2A). During the initial pandemic phase, these seasonal patterns largely disappeared, whereas new COVID-19 admissions were positively associated with observed fall rates, indicating heightened system strain during this phase. In the later phase, seasonal rhythms re-emerged, as evidenced by significant Fourier cosine terms, and the new COVID-19 burden remained positively associated with observed falls. In contrast, total COVID-19 inpatient counts showed an inverse association, suggesting potential changes in the system’s response over time.

#### 3.3.2. Expected Fall Rate

Expected fall rates demonstrated minimal seasonal fluctuation and no evident cyclic pattern (Figure 2B). During the initial pandemic phase, both new COVID-19 admissions and total COVID-19 inpatient counts were negatively associated with expected fall rates. In the later phase, no significant associations were observed, indicating that the patient case mix stabilized over time.

#### 3.3.3. Observed-to-Expected (O/E) Ratio

The O/E ratio closely mirrored the temporal patterns of observed fall rates, which is consistent with the relative stability of expected rates (Figure 2C). Seasonal effects were attenuated during the initial phase but re-emerged in the later phase, as indicated by significant Fourier sine and cosine terms. New COVID-19 admissions were consistently and positively associated with O/E ratios across all phases. In contrast, total COVID-19 inpatient counts shifted from a positive association in the initial phase to a significant negative association in the later phase, indicating a shift in the association between system burden and risk-adjusted fall outcomes over time.

### 3.4. Exploratory Temporal Associations Between Fall Rates and COVID-19 Burden

Time-lagged associations between new COVID-19 admissions and observed fall rates varied across pandemic phases.

**Figure 3:**
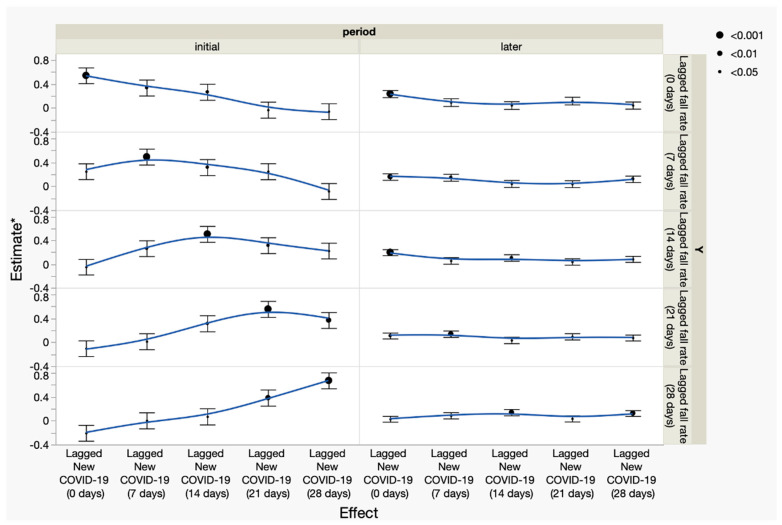
Synchronized temporal associations between fall rates and new COVID-19 admissions across lag intervals (0, 7, 14, 21, and 28 days), stratified by pandemic phase (initial and later). Estimates ± standard errors from lag-specific regression models are plotted for each phase, with significance levels represented by dot size and trends connected by smoothed lines. Associations were stronger and showed clearer lag-dependent patterns in the initial phase, but weakened and became less consistent in the later phase. *: Estimates are scaled ×1000 for clarity.

Figure 3 shows exploratory temporal associations between new COVID-19 admissions and inpatient fall rates across lag intervals ranging from 0 to 28 days. During the initial pandemic phase, positive associations were observed consistently across all examined lag intervals, whereas during the later phase, these associations were attenuated and less consistent across lag structures. These analyses were conducted to aid the interpretation of temporal patterns in fall outcomes. Full regression results are provided in Supplementary Table S2.

## 4. Discussion

This study examined longitudinal changes in inpatient fall outcomes during periods of large-scale system stress using a risk-adjusted benchmarking approach. Directly supported by our data, observed and expected fall rates increased during the pandemic, whereas the observed-to-expected (O/E) ratio declined only in the later phase. This pattern indicates that changes in crude fall rates and changes in risk-adjusted safety performance did not occur in parallel, underscoring the value of risk-adjusted surveillance for disentangling shifts in patient case mix from changes in system-level performance.

Previous studies conducted during the COVID-19 pandemic primarily reported higher inpatient fall rates based on crude incidence measures, which limit the interpretation of hospital safety performance under disrupted care conditions [11,12]. In contrast, our findings show that risk-adjusted indicators can capture phase-specific changes in safety performance that are not apparent from crude rates alone. This observation suggests that exclusive reliance on unadjusted fall rates may obscure important aspects of organizational performance during prolonged periods of system disruption.

Before the pandemic, inpatient fall outcomes demonstrated regular seasonal variations, consistent with prior research on hospital safety rhythms. Our descriptive and regression analyses indicate that these seasonal patterns were attenuated during the initial phase of the pandemic. Similar disruption of routine safety processes has been reported during early phases of large-scale healthcare emergencies [9,18]. An interpretation consistent with these observations is that acute system stress may temporarily disrupt established organizational rhythms related to patient safety.

Differences observed across pandemic phases likely reflect varying degrees of system strain over time. In Japan, the early pandemic period was marked by repeated emergency declarations and rapid organizational reconfiguration, including the admission of patients from the Diamond Princess cruise ship, which imposed substantial psychological and operational stress on healthcare staff [15,16]. Subsequent studies have suggested that such strain may persist beyond the initial crisis phase [8,10,19]. In contrast, later phases coincided with partial organizational adaptation and stabilization of care delivery under sustained pressure [20]. These contextual factors provide a plausible backdrop for interpreting phase-specific patterns in fall indicators, although causal mechanisms cannot be established from the present observational data.

In later phases, seasonal regularity in fall outcomes re-emerged. While this pattern cannot be directly attributed to specific organizational changes, it is consistent with partial stabilization of hospital operations over time. Similarly, the shift in associations between total COVID-19 inpatient burden and fall outcomes across phases may reflect evolving care organization or staff responses under sustained system pressure. These interpretations should be regarded as hypothesis-generating rather than confirmatory.

Exploratory temporal analyses further showed that associations between system burden indicators and fall outcomes were strongest during the initial phase and attenuated in later phases. These findings are consistent with the hypothesis that safety outcomes may be more tightly coupled to operational strain during periods of acute disruption, with weaker coupling as systems adapt. Comparable time-dependent associations between external stressors and health outcomes have been described in other contexts, such as delayed health effects following environmental exposures or extreme events [21,22]. In the present study, lag analyses were intended to support the interpretation of temporal patterns rather than to infer causal relationships.

Inpatient falls are widely recognized as nursing-sensitive safety indicators influenced by patient characteristics, care processes, and ward-level conditions [23]. Taken together, our findings indicate that risk-adjusted inpatient fall indicators may be useful for monitoring hospital safety performance under fluctuating care demands. From an organizational and clinical governance perspective, such indicators may support nursing leaders and hospital safety teams in interpreting safety trends during periods of systemic stress, including workforce shortages or seasonal demand surges, beyond pandemic contexts.

## 5. Limitations

This study has several limitations. First, the fall prediction model used to estimate expected rates was calibrated using pre-pandemic data and did not incorporate disruption-specific care factors; therefore, changes in care environments during the pandemic may not have been fully captured. Second, this single-center study conducted in a Japanese acute care hospital may reflect context-specific organizational characteristics, limiting generalizability to other healthcare settings. Third, the observational design and temporal analyses examined associations rather than causal relationships, and the absence of detailed operational and staffing data precluded examination of underlying mechanisms. Future multicenter studies incorporating higher-resolution operational data are needed to further refine risk-adjusted safety monitoring approaches.

## 6. Conclusions

This study examined temporal patterns in inpatient fall outcomes during periods of large-scale system stress using a risk-adjusted benchmarking approach. The findings suggest that incorporating O/E ratios can support the interpretation of inpatient fall trends by accounting for concurrent changes in patient risk profiles and care environments. In this cohort, although both observed and expected fall rates increased during the pandemic, declines in O/E ratios were observed in later phases, indicating phase-specific variation in risk-adjusted fall outcomes that was not fully captured by crude fall rates alone.

By applying a risk-standardized approach to a nursing-sensitive safety indicator, this study highlights the potential value of distinguishing intrinsic patient risk from system-level influences when evaluating inpatient safety. Rather than serving as an exhaustive indicator of overall health system performance, the O/E ratio may function as a complementary tool to support safety monitoring and assessment, particularly during periods of disrupted or fluctuating care demand.

Taken together, these findings indicate that risk-adjusted inpatient fall indicators may assist nursing leaders and hospital safety teams in more accurately contextualizing safety performance under conditions of systemic stress, such as pandemics or workforce disruptions. Further multicenter research is required to evaluate the generalizability of this approach and to clarify how risk-adjusted indicators can be integrated into broader hospital safety surveillance and clinical governance frameworks.

## Figures and Tables

**Table 1:** Summary statistics of observed fall rates, expected fall rates, and O/E ratios across pandemic phases.

Variable	Pre-Pandemic	Initial Pandemic	Later Pandemic	Tukey–Kramer Test (*p* < 0.05)
Observed fall rate (%)	1.63 ± 0.02	2.05 ± 0.02	2.12 ± 0.02	Initial > Pre; Later > Pre
Expected fall rate (%)	1.68 ± 0.01	2.12 ± 0.01	2.36 ± 0.01	Initial > Pre; Later > Initial; Later > Pre
O/E ratio	0.98 ± 0.01	0.97 ± 0.01	0.90 ± 0.01	Later < Pre; Later < Initial

Values are expressed as least squares means ± standard errors derived from 28-day moving average (MA) data analyzed with general linear models, including fixed effects for period and month. Post hoc Tukey–Kramer HSD tests were applied for pairwise comparisons among periods. “>” and “<” indicate *p* < 0.05.

**Table 2:** The linear regression results for fall outcomes by pandemic period (initial and later): the effects of COVID-19-related variables (simplified version).

Outcome	Effect	Initial		Later	
		Est (95% CI) *	*p* Value	Est (95% CI) *	*p* Value
	Intercept	19.12 (18.35–19.88)	<0.001	17.64 (−17.05–18.23)	<0.001
Observed fall rate	New COVID-19 admissions	1.28 (0.36–2.20)	<0.01	1.98 (1.71–2.26)	<0.001
	Total COVID-19 inpatients	0.09 (−0.11–0.29)	NS	−0.20 (−0.26–−0.14)	<0.001
	Intercept	22.38 (22.21–22.54)	<0.001	23.76 (23.53–23.98)	<0.001
Expected fall rate	New COVID-19 admissions	−0.39 (−0.58–−0.19)	<0.001	0.01 (−0.09–0.11)	NS
	Total COVID-19 inpatients	−0.16 (−0.20–−0.12)	<0.001	0.02 (−0.01–0.04)	NS
	Intercept	0.84 (0.81–0.88)	<0.001	0.72 (0.70–0.75)	<0.001
O/E ratio	New COVID-19 admissions	0.08 (0.03–0.12)	<0.001	0.09 (0.08–0.10)	<0.001
	Total COVID-19 inpatients	0.01 (0.00–0.02)	<0.01	−0.01 (−0.01–−0.01)	<0.001

Values are expressed as regression coefficients with 95% confidence intervals (CIs). Only COVID-19–related variables (new admissions and total inpatients) are shown. Full results, including month and seasonal terms, are provided in Supplementary Table S1. Lag analyses are reported separately in Supplementary Table S2. *: Estimates and 95% confidence interval limits for observed and expected fall rates are scaled ×1000 for clarity. Accordingly, regression coefficients should be interpreted as changes per 1000 units of the outcome.

## Data Availability

The datasets presented in this article are not readily available because the data are part of an ongoing study. Requests to access the datasets should be directed to the corresponding author.

## Supplementary Materials

The following supporting information can be downloaded at: https://www.mdpi.com/article/10.3390/healthcare14030358/s1, Table S1: Linear regression results for fall outcomes by pandemic period (Pre, Initial, Later): Full model including month and seasonal terms; Table S2: Exploratory regression results for temporal associations between inpatient fall rates and new COVID-19 admissions across lag intervals (0–28 days).

## Abbreviations

The following abbreviations are used in this manuscript:

COVID-19	Coronavirus disease 2019
O/E	Observed-to-expected
OLS	Ordinary least squares
