# Supplementary material for: Risk-Adjusted Inpatient Falls as Indicators of Health System Performance During the COVID-19 Pandemic
Source: Healthcare (Basel). 2026 Jan 30;14(3):358. doi: 10.3390/healthcare14030358 (PMC12897820; doi:10.3390/healthcare14030358)
Supplement: Supplementary file 1 [file healthcare-14-00358-s001.zip › healthcare-4094949-supplementary.pdf]

Supplemental Table S1. Linear regression results for fall outcomes by pandemic period (Pre, Initial, Later): Full model including month and seasonal terms

		Pre#					Initial					Later				
**Fall rate and O/E**	**Effect**	**Estimate ***	**Lower 95% ***	**Upper 95%***	**t value**	**p value**	**Estimate ***	**Lower 95% ***	**Upper 95%***	**t value**	**p value**	**Estimate ***	**Lower 95% ***	**Upper 95%***	**t value**	**p value**
	Intercept	16.31	16.07	16.55	133.36	<0.001	19.12	18.35	19.88	49.33	<0.001	17.64	17.05	18.23	59.01	<0.001
	New COVID-19 admissions	NC	NC	NC	NC	NS	1.28	0.36	2.20	2.74	<0.01	1.98	1.71	2.26	14.29	<0.001
	Total COVID-19 inpatients	NC	NC	NC	NC	NS	0.09	-0.11	0.29	0.88	NS	-0.20	-0.26	-0.14	-6.55	<0.001
	Mon [Jan]	-2.60	-4.97	-0.22	-2.15	<0.05	0.98	-2.44	4.41	0.56	NS	-1.32	-3.93	1.30	-0.99	NS
	Mon [Feb]	1.56	-0.77	3.90	1.32	NS	-3.09	-6.39	0.21	-1.84	NS	-4.47	-7.10	-1.84	-3.33	<0.001
	Mon [Mar]	-0.03	-2.25	2.19	-0.03	NS	2.05	-1.23	5.33	1.23	NS	-4.53	-7.11	-1.94	-3.44	<0.001
	Mon [Apr]	-1.25	-3.45	0.96	-1.11	NS	0.79	-2.40	3.98	0.49	NS	2.45	-0.27	5.16	1.77	NS
	Mon [May]	5.42	3.11	7.73	4.61	<0.001	-0.60	-3.70	2.50	-0.38	NS	5.00	2.24	7.76	3.56	<0.001
**Fall rate (Observed)**	Mon [Jun]	-0.42	-2.87	2.02	-0.34	NS	0.32	-2.78	3.42	0.20	NS	6.94	4.14	9.74	4.86	<0.001
	Mon [Jul]	-0.55	-3.02	1.92	-0.44	NS	-1.78	-4.95	1.39	-1.11	NS	4.76	2.02	7.51	3.41	<0.001
	Mon [Aug]	4.42	2.06	6.79	3.67	<0.001	1.90	-1.39	5.18	1.13	NS	-8.25	-11.26	-5.25	-5.40	<0.001
	Mon [Sep]	-4.25	-6.50	-2.00	-3.71	<0.001	0.25	-3.09	3.58	0.15	NS	2.78	0.00	5.57	1.96	NS
	Mon [Oct]	-5.13	-7.34	-2.93	-4.57	<0.001	0.01	-3.38	3.40	0.01	NS	1.94	-0.72	4.59	1.43	NS
	Mon [Nov]	4.88	2.56	7.19	4.15	<0.001	5.37	2.09	8.64	3.22	<0.01	-0.20	-2.88	2.48	-0.15	NS
	Mon [Dec]	-2.05	-4.42	0.31	-1.71	NS	-6.19	-9.42	-2.97	-3.77	<0.001	-5.11	-7.75	-2.46	-3.79	<0.001
	Fourier_Cos	0.24	-2.01	2.49	0.21	NS	0.39	-2.58	3.37	0.26	NS	3.41	0.84	5.97	2.61	<0.01
	Fourier_Sin	0.69	-1.47	2.84	0.63	NS	-2.02	-5.17	1.12	-1.26	NS	2.14	-0.38	4.65	1.67	NS
	Intercept	16.47	16.38	16.57	336.64	<0.001	22.38	22.21	22.54	270.36	<0.001	23.76	23.53	23.98	210.50	<0.001
	New COVID-19 admissions	NC	NC	NC	NC	NS	-0.39	-0.58	-0.19	-3.87	<0.001	0.01	-0.09	0.11	0.16	NS
	Total COVID-19 inpatients	NC	NC	NC	NC	NS	-0.16	-0.20	-0.12	-7.45	<0.001	0.02	-0.01	0.04	1.46	NS
	Mon [Jan]	1.58	0.63	2.53	3.27	<0.01	2.83	2.10	3.56	7.61	<0.001	-0.24	-1.23	0.74	-0.48	NS
	Mon [Feb]	-0.09	-1.03	0.84	-0.19	NS	-0.24	-0.94	0.47	-0.66	NS	-1.97	-2.96	-0.97	-3.89	<0.001
	Mon [Mar]	-1.03	-1.92	-0.14	-2.28	<0.05	-0.56	-1.26	0.14	-1.57	NS	-2.61	-3.59	-1.63	-5.25	<0.001
	Mon [Apr]	-0.88	-1.76	0.01	-1.95	NS	0.31	-0.37	0.99	0.89	NS	1.73	0.70	2.75	3.32	<0.001
	Mon [May]	1.04	0.12	1.97	2.22	<0.05	0.72	0.06	1.38	2.13	<0.05	3.40	2.36	4.44	6.41	<0.001
**Fall rate (Expected)**	Mon [Jun]	0.75	-0.23	1.73	1.51	NS	0.11	-0.55	0.77	0.32	NS	1.07	0.01	2.12	1.98	<0.05
	Mon [Jul]	0.55	-0.43	1.54	1.10	NS	-1.50	-2.18	-0.82	-4.35	<0.001	2.44	1.41	3.48	4.63	<0.001
	Mon [Aug]	-0.28	-1.22	0.67	-0.58	NS	-0.35	-1.05	0.35	-0.97	NS	-0.21	-1.35	0.92	-0.37	NS
	Mon [Sep]	-1.49	-2.39	-0.59	-3.25	<0.01	-0.65	-1.36	0.06	-1.79	NS	-0.57	-1.62	0.48	-1.06	NS
	Mon [Oct]	-3.49	-4.37	-2.61	-7.78	<0.001	-0.81	-1.54	-0.09	-2.21	<0.05	-1.06	-2.06	-0.05	-2.07	<0.05
	Mon [Nov]	2.39	1.46	3.31	5.08	<0.001	-0.78	-1.48	-0.08	-2.19	<0.05	-1.06	-2.07	-0.05	-2.06	<0.05
	Mon [Dec]	0.94	-0.01	1.88	1.95	NS	0.92	0.23	1.61	2.62	<0.01	-0.92	-1.92	0.08	-1.82	NS
	Fourier_Cos	0.49	-0.41	1.40	1.08	NS	0.35	-0.28	0.99	1.09	NS	0.31	-0.66	1.27	0.62	NS
	Fourier_Sin	0.61	-0.25	1.48	1.40	NS	-1.34	-2.01	-0.66	-3.91	<0.001	-0.46	-1.41	0.49	-0.95	NS
	Intercept	0.99	0.97	1.00	141.22	<0.001	0.84	0.81	0.88	44.59	<0.001	0.72	0.70	0.75	53.61	<0.001
	New COVID-19 admissions	NC	NC	NC	NC	NS	0.08	0.03	0.12	3.44	<0.001	0.09	0.08	0.10	14.68	<0.001
	Total COVID-19 inpatients	NC	NC	NC	NC	NS	0.01	0.00	0.02	2.69	<0.01	-0.01	-0.01	-0.01	-7.13	<0.001
	Mon [Jan]	-0.24	-0.37	-0.10	-3.44	<0.001	-0.08	-0.24	0.09	-0.89	NS	-0.08	-0.19	0.04	-1.26	NS
	Mon [Feb]	0.10	-0.03	0.23	1.46	NS	-0.13	-0.29	0.03	-1.57	NS	-0.14	-0.26	-0.02	-2.28	<0.05
	Mon [Mar]	0.05	-0.07	0.18	0.83	NS	0.15	-0.01	0.31	1.86	NS	-0.12	-0.23	0.00	-1.95	NS
	Mon [Apr]	-0.02	-0.15	0.10	-0.34	NS	0.04	-0.11	0.20	0.53	NS	0.06	-0.07	0.18	0.92	NS
	Mon [May]	0.27	0.14	0.40	3.98	<0.001	-0.05	-0.20	0.10	-0.61	NS	0.14	0.02	0.26	2.21	<0.05
**O/E ratio**	Mon [Jun]	-0.05	-0.19	0.09	-0.75	NS	0.01	-0.14	0.16	0.09	NS	0.31	0.18	0.44	4.79	<0.001
	Mon [Jul]	-0.05	-0.19	0.10	-0.64	NS	-0.02	-0.18	0.13	-0.29	NS	0.13	0.01	0.26	2.10	<0.05
	Mon [Aug]	0.32	0.19	0.46	4.70	<0.001	0.10	-0.06	0.26	1.18	NS	-0.37	-0.51	-0.24	-5.38	<0.001
	Mon [Sep]	-0.19	-0.32	-0.06	-2.88	<0.01	0.01	-0.15	0.18	0.16	NS	0.13	0.01	0.26	2.08	<0.05
	Mon [Oct]	-0.14	-0.27	-0.01	-2.18	<0.05	0.02	-0.15	0.18	0.22	NS	0.12	0.00	0.24	1.96	NS
	Mon [Nov]	0.12	-0.01	0.25	1.81	NS	0.28	0.12	0.44	3.45	<0.001	0.02	-0.11	0.14	0.26	NS
	Mon [Dec]	-0.18	-0.31	-0.04	-2.61	<0.01	-0.34	-0.49	-0.18	-4.19	<0.001	-0.21	-0.33	-0.09	-3.39	<0.001
	Fourier_Cos	0.00	-0.13	0.13	-0.03	NS	0.01	-0.13	0.16	0.16	NS	0.17	0.06	0.29	2.90	<0.01
	Fourier_Sin	0.02	-0.11	0.14	0.27	NS	-0.06	-0.21	0.09	-0.77	NS	0.12	0.01	0.24	2.14	<0.05

*: Estimates and 95% confidence interval limits are scaled × 1,000 for clarity.
#: No new COVID-19 admissions occurred in the pre-pandemic period; regression coefficients were not estimated (NC).
The full model included COVID-19–related variables (new admissions and total inpatients), month indicator variables, and seasonal Fourier terms.
This table provides complete results corresponding to the simplified model shown in Table 2 of the main text.

Supplemental Table S2. Exploratory regression results for temporal associations between inpatient fall rates and new COVID-19 admissions across lag intervals (0–28 days)

Y	Effect	Initial				Later			
		Estimate*	SEM*	t value	p value	Estimate*	SEM*	t value	p value
Lagged fall rate (0 days)	Intercept	19.711	0.261	75.51	<0.001	19.488	0.247	78.84	<0.001
	Lagged New COVID-19 (0 days)	0.541	0.131	4.13	<0.001	0.236	0.060	3.95	<0.001
	Lagged New COVID-19 (7 days)	0.337	0.133	2.53	<0.05	0.093	0.064	1.45	NS
	Lagged New COVID-19 (14 days)	0.266	0.134	1.99	<0.05	0.044	0.065	0.68	NS
	Lagged New COVID-19 (21 days)	-0.033	0.133	-0.25	NS	0.119	0.063	1.87	NS
	Lagged New COVID-19 (28 days)	-0.058	0.132	-0.44	NS	0.044	0.059	0.74	NS
Lagged fall rate (7 days)	Intercept	19.487	0.261	74.55	<0.001	19.781	0.224	88.13	<0.001
	Lagged New COVID-19 (0 days)	0.249	0.131	1.90	NS	0.160	0.054	2.94	<0.01
	Lagged New COVID-19 (7 days)	0.493	0.133	3.70	<0.001	0.149	0.058	2.55	<0.05
	Lagged New COVID-19 (14 days)	0.318	0.134	2.37	<0.05	0.045	0.059	0.76	NS
	Lagged New COVID-19 (21 days)	0.249	0.134	1.86	NS	0.041	0.058	0.71	NS
	Lagged New COVID-19 (28 days)	-0.082	0.133	-0.62	NS	0.122	0.054	2.27	<0.05
Lagged fall rate (14 days)	Intercept	19.367	0.262	74.00	<0.001	20.024	0.211	94.83	<0.001
	Lagged New COVID-19 (0 days)	-0.046	0.131	-0.35	NS	0.199	0.051	3.91	<0.001
	Lagged New COVID-19 (7 days)	0.267	0.133	2.00	<0.05	0.059	0.055	1.08	NS
	Lagged New COVID-19 (14 days)	0.509	0.134	3.79	<0.001	0.109	0.055	1.97	<0.05
	Lagged New COVID-19 (21 days)	0.317	0.134	2.37	<0.05	0.042	0.054	0.77	NS
	Lagged New COVID-19 (28 days)	0.226	0.133	1.70	NS	0.086	0.050	1.70	NS
Lagged fall rate (21 days)	Intercept	19.384	0.262	74.09	<0.001	20.268	0.205	98.93	<0.001
	Lagged New COVID-19 (0 days)	-0.102	0.131	-0.78	NS	0.111	0.049	2.23	<0.05
	Lagged New COVID-19 (7 days)	0.016	0.133	0.12	NS	0.141	0.053	2.65	<0.01
	Lagged New COVID-19 (14 days)	0.315	0.134	2.35	<0.05	0.036	0.054	0.68	NS
	Lagged New COVID-19 (21 days)	0.556	0.134	4.16	<0.001	0.097	0.053	1.85	NS
	Lagged New COVID-19 (28 days)	0.370	0.133	2.79	<0.01	0.077	0.049	1.57	NS
Lagged fall rate (28 days)	Intercept	19.523	0.263	74.17	<0.001	20.487	0.201	102.06	<0.001
	Lagged New COVID-19 (0 days)	-0.204	0.132	-1.55	NS	0.030	0.048	0.61	NS
	Lagged New COVID-19 (7 days)	0.004	0.134	0.03	NS	0.088	0.052	1.69	NS
	Lagged New COVID-19 (14 days)	0.071	0.135	0.52	NS	0.139	0.053	2.65	<0.01
	Lagged New COVID-19 (21 days)	0.384	0.134	2.86	<0.01	0.037	0.052	0.71	NS
	Lagged New COVID-19 (28 days)	0.672	0.134	5.03	<0.001	0.126	0.048	2.62	<0.01

*:Values for estimates and standard errors (SEM) are scaled ×1000 for clarity.
